# How Attribution of COVID-19 Crisis Responsibility Predicts Hong Kong Citizens’ Intention to Accept Vaccination †

**DOI:** 10.3390/vaccines12121305

**Published:** 2024-11-22

**Authors:** Ji Won Kim, Qinxian Cai, Lang Kao, Yi-Hui Christine Huang

**Affiliations:** 1 Department of Media and Communication, City University of Hong Kong, Kowloon Tong, Hong Kong; jiwonkim@cityu.edu.hk (J.W.K.); yihhuang@cityu.edu.hk (Y.-H.C.H.); 2 Department of Social Science, The Hang Seng University, New Territories, Hong Kong; lkao@hsu.edu.hk

**Keywords:** crisis responsibility attribution, trust, anger, vaccination intention, the pandemic

## Abstract

Background: This study aims to illuminate the role of perceived crisis responsibility in shaping vaccination intention. By using the case of Hong Kong during the COVID-19 pandemic, we examined whether and how the allocation of crisis responsibility to the government predicts the public’s intention to take vaccines, particularly by investigating its underlying mechanism. Method and Results: Based on a population-representative sample of Hong Kong adults (*N* = 3188), our results indicated that (1) the attribution of crisis responsibility directly led to lower vaccination intention, and (2) it also had indirect influences on vaccination intention through trust and anger; specifically, the crisis attribution resulted in less willingness to take vaccines via a decreased trust in government health agencies. We also found a serial mediation pathway in which anger aroused by the crisis attribution could decrease trust, which, in turn, yielded lower vaccination intentions. Conclusion: The findings of this study offer theoretical insights into the role of attribution of crisis responsibility in affecting vaccination decisions during a public health emergency. Further, these findings provide directions for crisis managers and public health authorities to develop communication strategies to motivate vaccine uptake and formulate an approach to tackle the pandemic crisis.

## 1. Introduction

During a public health emergency, the public’s cooperation with the government’s health promotion efforts is crucial to tackling the crisis. In the COVID-19 pandemic, following the introduction and distribution of COVID-19 vaccines, governments actively encouraged their citizens to get vaccinated to curb the spread of the virus. However, public skepticism over the safety and efficacy of COVID-19 vaccines continued throughout vaccine administration [1,2].

While extant literature has explored the antecedents of COVID-19 vaccination acceptance, such as socio-psychological factors and perceptions of vaccine-related characteristics, scant research has focused on the role that the public’s judgment surrounding the pandemic might play in affecting their vaccination decisions [3,4,5,6]. In times of crisis, people’s interpretation of the crisis—how they place responsibility for the crisis—may determine their reactions [7]. In the literature on crisis communication, responsibility attribution has been identified as a crucial factor in shaping the public response to a crisis by influencing their views on the issue and the organizations involved in the crisis [8,9]. In the context of a public health crisis, the government is typically the central actor in managing a crisis, making it the focal point of responsibility. Nevertheless, less is known about whether and to what extent the allocation of crisis responsibility could have a bearing on the public’s compliance with health-protective actions.

In Hong Kong, the government’s handling of the pandemic was met with criticism—for instance, the government’s refusal to close borders with mainland China in the early phase of the pandemic caused a public backlash against its decision, and Hong Kong residents decried the shortage of masks and personal protective equipment [10]. Furthermore, despite the Hong Kong government’s early rollout of COVID-19 vaccines and their active push for vaccinations, they experienced difficulty getting residents to participate in the vaccination drive [11,12]. In this regard, the investigation of the dynamics of Hong Kong residents’ reactions to the pandemic will help disentangle the mechanism that underlies the influence of crisis attribution on pro-health behaviors during a global health crisis, which we believe could provide broader implications for examining this process across contexts and further informing future crises.

The primary purpose of the current study is to illuminate the role of governmental responsibility in determining Hong Kong residents’ responses to the pandemic and their uptake of health-protective action—specifically, their intention to get vaccinated against COVID-19. We first aim to examine the impact of perceived crisis responsibility on the acceptance of COVID-19 vaccines as well as trust in government health agencies. Guided by the theoretical frameworks of Weiner’s attribution theory (1995) and the situational crisis communication theory (SCCT) [7,13], we seek to examine how the degree to which Hong Kong’s public assigns the responsibility of pandemic management to the government could predict their trust in government agencies in charge of disease prevention, and whether this would, in turn, hinder their willingness to accept COVID-19 vaccines.

Second, this study aims to investigate the role of the affective mechanism that may account for the impact of crisis responsibility on vaccine acceptance. By integrating theoretical perspectives from emotional psychology—particularly, the functional emotion theory—and the communication literature on attribution and crisis emotions, we seek to unpack the processes by which the public’s felt anger would mediate the impact of crisis responsibility on their intention to adopt vaccinations [14,15,16].

We expect that the results of this study will contribute to developing a theoretical model that provides insights into the psychological mechanisms underpinning the effects of crisis responsibility on risk-related judgment and decision-making during a public health emergency. As one of the few studies to apply the theoretical framework of attributed responsibility to understanding public decisions related to health and risk behaviors, this study intends to shed light on the underexplored role of crisis responsibility in promoting pro-health outcomes by identifying the pathway through which crisis responsibility predicts vaccination intention and synthesizing theoretical explanations grounded in interdisciplinary studies about health communication, crisis communication, and emotional psychology. Particularly, we aim to delve deeper into the cognitive and affective routes to persuading the public to accept vaccinations—via trust in an organization and anger—to elucidate the mediating roles of these factors. Furthermore, this study has managerial and practical implications for communication professionals and health campaigners in terms of developing communication strategies to motivate the uptake of health-protective actions and the acceptance of organizational efforts to control crises in a time when there is a growing number of global health emergencies.

## 2. Literature Review

### 2.1. Attribution of Crisis Responsibility in a Pandemic: Victim or Assailant?

The attribution of crisis responsibility has been identified as a key determinant for shaping the public’s perception of and their behaviors toward an organization involved in a crisis [9]. As one of the major frameworks developed and employed to study the relationship between an organization and stakeholders’ responses during crises, situational crisis communication theory (SCCT) offers a crucial theoretical lens for understanding how stakeholders interpret crisis situations and assess organizational responsibility for these situations, which could affect their reactions to the organization [8].

The tenets of the SCCT are rooted in Weiner’s attribution theory in the area of social psychology [8,17]. Attribution theory suggests that individuals are motivated to find the reasons behind events; when they are faced with unexpected or negative events, they are likely to assign responsibility for those events [13,17]. This theory proposes that the attribution of responsibility occurs when there is an identifiable source of an action, and people believe that the source should have predicted or controlled the outcome of a particular event they experience [13]. SCCT extends this process of attribution to the realm of crisis management, which further argues that the type of crisis faced by an organization can be determined depending on the level of crisis responsibility attribution [7]. Specifically, when people view the organization as having very weak responsibility for the crisis event, this type of crisis can be identified as a *victim cluster*. On the other hand, if the organization is deemed responsible for the crisis at a moderate level, it is categorized as an *accidental cluster*. If the organization is seen as knowingly putting people at risk and being responsible for the crisis at a strong level, this situation is defined as a *preventable* (*intentional*) *cluster* [8]. 

The COVID-19 pandemic provides communication researchers with an intriguing example for examining how the attribution of crisis responsibility may shape public judgment of a government organization and decisions on the acceptance of pro-health behaviors. First and foremost, assessing the crisis type of this pandemic is key to understanding the public perception surrounding it. Whether it is viewed purely as a natural disaster or a human-error crisis is an important concern. While it remains debatable whether the pandemic outbreak was caused by acts of nature or by (accidental or preventable) human acts (e.g., laboratory leak of the virus, bioengineered weapon), the offset responsibility (i.e., responsible for the continuation and management of the problem) of the pandemic could have played out when it comes to forming people’s perceptions of this crisis [13,18]. For instance, surveys found that the public’s evaluation of the government’s handling of the pandemic was closely tied to their attitudes toward the pandemic and health-protective actions [19,20]. It is therefore likely that the public might have considered the pandemic a *preventable* crisis, and as they ascribed a strong responsibility to the government for the mismanagement of the situation, it could affect their acceptance of protective behaviors like vaccinations. Nevertheless, its potential role—whether it could have a direct influence on pro-health outcomes—has not yet been investigated. We raise the following research question.

**Research Question 1**: Will the attribution of COVID-19 crisis responsibility be associated with the intention to receive COVID-19 vaccines? 

### 2.2. Crisis Attribution and Trust

The degree of trust in an organization has been recognized as one of the major resources that should be retained, as it determines the public’s willingness to engage in communication with an organization during a crisis and their support for organizational goals to contain the crisis [16,21]. In general terms, trust is defined as a psychological state of accepting vulnerability based upon positive expectations of the intentions or actions of another party [22]. Trust does not involve risk per se, but it implies “a willingness to engage in risk-taking with the focal party” [23] (p. 124). High trust can result in the decision to cooperate, share information with the trustee, and willingly allow the trustee’s control over issues that are significant to the trustor [23].

While there are several integrative models of trust across disciplines, public trust in an organization is largely conceptualized in terms of three major aspects—ability, benevolence, and integrity [23]. Ability refers to the capability of an organization to perform its jobs (knowledge and skills to do well) [24]. Benevolence refers to the degree to which an organization is believed to do good to the public (concerns or cares for the public’s interest) [24]. Integrity pertains to the belief that an organization adheres to a set of ethical standards (being fair and just) [24,25].

Prior research has demonstrated that the attribution of crisis responsibility can have a significant impact on the public’s perception of an organization [9,26,27]. For example, a previous study found that Hong Kong consumers’ judgments of the organizational responsibility for an airline crash incident resulted in lower trust in the organization [26]. In a meta-analysis of SCCT research, it showed that the attribution of organizational crisis responsibility was negatively associated with organizational reputation [9]. Relatedly, Nekmat and Kong reported that the attribution of organizational crisis responsibility led to a more negative attitude toward the organization [27]. Extending these findings to the context of the COVID-19 pandemic, we postulate that the attribution of COVID-19 crisis responsibility might negatively predict trust in managing government organizations. That is, those who allocate greater responsibility for the COVID-19 crisis to the government would be less likely to trust government health agencies in charge of managing disease control and prevention.

**Hypothesis** **1** **(H1).** *Attribution of COVID-19 crisis responsibility will be negatively associated with trust in government health agencies.*

Scholars also underscored the crucial role of trust in affecting the public’s behavioral responses. Trust in an organization can serve as a key predictor of individuals’ behavioral responses, such as cooperation and compliance with an organization [28,29]. Studies have found that a higher degree of trust in an organization yields a greater intention to engage in supportive behaviors toward the organization [30,31]. Relevant to the context of health crises, a COVID-19 study showed that trust in a government during the COVID-19 pandemic led to positive Word-of-Mouth (WOM) intention to vaccinate [32]. Building upon these findings on the link between public trust and their behavioral responses, we anticipate the following:

**Hypothesis** **2** **(H2).** *Trust in government health agencies will be positively associated with the intention to receive COVID-19 vaccines.*

### 2.3. Crisis Attribution and Anger

Along with the degree of trust in an organization, another crucial aspect that needs to be considered in crises is an affective component—emotions that people experience in response to crises. Communication scholars have addressed the need to investigate the role of emotions in influencing the public’s cognitive and behavioral responses to crises [33,34,35]. Particularly, emotions triggered by crises have been examined from an attribution perspective [33]. This line of research is built upon Weiner’s model of attribution-emotion. According to Weiner’s model, emotions derive from people’s interpretations of an event [17]. When the outcome of an event is perceived to be negative, unexpected, or important, people seek to find the cause of the outcome, which can elicit their affective responses [17]. 

Applying this proposition to crises, a stream of research has identified emotions salient in crises and investigated how they are related to the attribution of crisis responsibility [16,33,36]. Coombs and Holladay found that the attribution of crisis responsibility can generate stronger negative emotions, and primarily, anger tends to intensify when people ascribe greater crisis responsibility to an organization [33]. Relatedly, Choi and Lin showed that the perception of crisis responsibility was positively associated with reports of emotions such as anger and contempt [36].

Anger has been identified as one of the dominant emotions that people experience in crises [34]. Scholars claim that anger is essentially a judgmental emotion that derives from the perception of an offense [37]. Anger is induced by circumstances in which a demeaning offense against “me” and “mine’ is believed to have occurred [37,38]. In crises, people tend to feel anger when they encounter an offense from an organization that threatens their well-being [15]. There is usually an issue of blaming involved in this appraisal of the situation; the organization becomes an object of blame if people perceive that the organization is responsible for the harmful actions that could have been controlled or prevented [15,39]. During the COVID-19 pandemic, when people held the government accountable for the consequences that they believed could have been avoided, they could feel greater feelings of anger. Hence, we propose the following:

**Hypothesis** **3** **(H3).** *Attribution of COVID-19 crisis responsibility will be positively associated with anger.*

Furthermore, anger has been shown to generate the public’s negative perception of an organization [16,40,41]. Kim and Cameron found that participants exposed to anger-inducing crisis news exhibited more negative attitudes toward the company than those exposed to sadness-inducing news [40]. Utz et al. showed that anger elicited in a crisis scenario like a nuclear disaster resulted in a lower organizational reputation [41]. Research has also indicated that anger can negatively influence trust in an organization [16]. Kim and Niederdeppe found that the anger that students experienced during an influenza outbreak on a college campus generated a lower degree of trust in the college health center [16]. Given the above discussion on the link between anger and the perception of an organization, we hypothesize the following:

**Hypothesis** **4** **(H4).** *Anger will be negatively associated with trust in government health agencies.*

Scholars also argue that emotions can directly predict behavioral outcomes [35]. Emotions are considered to motivate individuals’ behaviors [42]. The functional emotion theory posits that when an emotion is evoked, it could generate a particular “action tendency” associated with the emotion that guides subsequent actions [37,38,43]. This theory suggests that discrete emotions can trigger different types of action tendencies, which predispose people to act in particular ways to deal with the problems that elicit emotions [42].

Specifically, anger has been associated with attack/reject action tendencies intended to eliminate perceived obstacles [14]. When people experience anger, it can show them that something is wrong in the environment, which in turn would motivate them to remove obstacles that could threaten their well-being [37]. To defend themselves, angry people would likely attack the source held to be blamed for the offense or reject messages from the blameworthy source [14]. Hence, it is conceivable that anger aroused by the attribution of crisis responsibility might lead to the repudiation of recommended actions by a responsible organization. Taken together, guided by the above discussions on the role of anger in eliciting attack/reject action tendencies, we expect that anger felt in the COVID-19 crisis would predict less willingness to accept the recommended protective actions (i.e., getting vaccinated against COVID-19).

**Hypothesis** **5** **(H5).** *Anger will be negatively associated with the intention to receive COVID-19 vaccines.*

**Figure 1:**
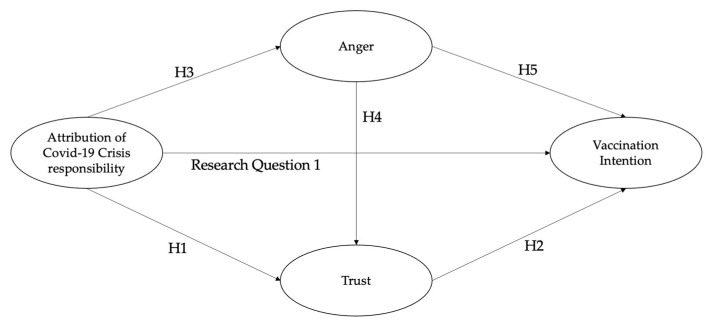
The hypothesized model for examining the effect of attribution of Covid-19 crisis responsibility on vaccination intention. Note. H6: Attribution of COVID-19 crisis responsibility → Trust in government health agencies → Vaccination intention; H7: Attribution of COVID-19 crisis responsibility → Anger → Vaccination intention; H8: Attribution of COVID-19 crisis responsibility → Anger → Trust in government health agencies → Vaccination intention.

A graphic illustration of the hypothesized model is shown in Figure 1. In addition to the direct paths discussed above, the hypothesized model involves the indirect paths that formulate the mechanisms by which the attribution of crisis responsibility leads to vaccination intention. Specifically, we examine whether the attribution of COVID-19 crisis responsibility would predict lower intentions to receive COVID-19 vaccines via decreased trust in government health agencies (**H6:** attribution of crisis responsibility → trust → vaccination intention). We also examine whether it would predict lower vaccination intention via greater anger (**H7**: attribution of crisis responsibility → anger → vaccination intention). Based on the positive relationship between anger and trust that is hypothesized above, we investigate the serial mediation path from the attribution of responsibility to vaccination intention via anger and trust (**H8**: attribution of crisis responsibility → anger → trust → vaccination intention).

## 3. Method

### 3.1. Survey Procedure and Participants

Data (*N* = 3188) was collected in Hong Kong using the survey panel in Rakuten Insights (Hong Kong) from September 2020 to October 2020. The survey was administered online, and potential participants were randomly drawn from the company’s online panel. Stratified quota sampling was adopted to recruit participants according to gender, age, and residence distribution in the Hong Kong population census. Population weights were also used in the data analysis to adjust the sample to the census data. All procedures for this study were approved by the University’s Institutional Review Board.

A total of 56.6% of respondents were female, whereas 43.4% of them were male. In total, 9.1% of them were between 20 and 24 years old, 11.5% were between 25 and 29 years old, 13% were between 30 and 34 years old, 12.9% were between 35 and 39 years old, 11.8% were between 40 and 44 years old, 8.9% were between 45 and 49 years old, 12% were between 50 and 54 years old, and 20.7% were over 55 years old. Regarding the education level, 25.3% completed a high school diploma, 3.5% had less than a high school diploma, 5.1% had a foundation diploma, 13.2% had an advanced diploma, 40.8% had a bachelor’s degree, 11.2% had a master’s degree, and 0.9% had a doctoral degree. In terms of the monthly household income, 22.6% reported above HK$60,000, 11.9% reported HK$50,001–HK$60,000, 17.1% reported HK$40,001–HK$50,000, 19.5% reported HK$30,001–HK$40,000, 15.1% reported HK$20,001–HK$30,000, 10.8% reported HK$10,001–HK$20,000, and 3% reported under HK$10,000. Regarding political stance, 10.3% identified themselves as Pro-Establishment, 12.8% as Centrist, 22.9% as Democrat, 10.2% as Nativist, 39.1% as Apolitical, and 4.8% as others (see Table 1). 

### 3.2. Measures

*Attribution of COVID-19 crisis responsibility*. On a seven-point scale ranging from one (no responsibility) to seven (all the responsibility), the attribution of crisis responsibility was assessed by asking how much responsibility (a) the central government and the (b) Hong Kong Special Administrative Region (HKSAR) government should bear the consequences of the COVID-19 pandemic. The central government refers to the government of the People’s Republic of China, which holds sovereignty over Hong Kong, while the HKSAR government operates as a semi-autonomous region under the “one country, two systems” framework, with its own governance structure in areas such as public health. Given the specific structure of the Hong Kong government system, we asked respondents to indicate how much responsibility they think different government entities (i.e., the central government, HKSAR government) should bear in handling the COVID-19 crisis (*M* = 5.29, *SD* = 1.39, *r* = 0.74, *p* < 0.001). 

*Trust in government health agencies*. Adapted from prior research, a 15-item scale was used to measure three dimensions of trust in government health agencies: ability, benevolence, and integrity [23,24]. We asked respondents to indicate how much they agree with the statements regarding government health agencies (1 = strongly disagree, 7 = strongly agree). Ability was measured by five items, such as “They are capable of performing their jobs” and “They have much knowledge about the work that needs to be done.” Benevolence was measured by five items, such as “They are very concerned about my welfare” and “They would not knowingly do anything to hurt me”. Integrity was measured by five items, such as “They try hard to be fair in dealing with others” and “They have a strong sense of justice.” (*M* = 4.05, *SD* = 1.32, α = 0.98).

*Anger*. On a seven-point scale (1 = strongly disagree, 7 = strongly agree), respondents were asked to indicate how much feelings of anger (i.e., angry, annoyed) they experienced during the COVID-19 pandemic (*M* = 4.46, *SD* = 1.51, *r* = 0.82, *p* < 0.001).

*Intention to accept vaccination*. On a seven-point scale (1 = strongly disagree, 7 = strongly agree), we assessed respondents’ intention to receive COVID-19 vaccines with three items. Items included “I would get the COVID-19 vaccine sometime soon”, “If I were faced with the decision of whether to get the COVID-19 vaccine today, I would choose to get it”, and “I would get the COVID-19 vaccine in the future” (*M* = 3.88, *SD* = 1.43, α = 0.88).

*Control variables*. Several control variables—gender, age, education level, monthly household income, and political stance—were considered and included, given their significant relationships with the variables of interest.

### 3.3. Data Analysis Procedure

In Table 2, we provide the descriptive statistics (mean and standard deviation) for the main variables in this study, along with their correlation results. The correlations between these variables are all significant, which supports proceeding with the subsequent structural equation modeling (SEM) analysis. To examine the hypothesized relationships within the model, SEM was conducted using AMOS 26.0 software. The model fit was assessed using the criteria recommended by Hu and Bentler [44].

## 4. Results

### 4.1. Model Fitting

Before testing the relationships in the hypothesized model, we performed a confirmatory factor analysis (CFA) to validate the measurement model. First, we tested the dimensionality of the scale of *trust in government health agencies*. The first-order single factor model showed a good fit to the data; χ^2^[56] = 275.473, CFI = 0.997, TLI = 0.994, RMSEA = 0.035, and SRMR = 0.009. This result provided support for the existence of the unidimensional structure. We conducted the CFA to verify the fit of the overall measurement model. The results indicated that the measurement model fitted the data well (χ^2^[169] = 709.616, CFI = 0.993, TLI = 0.991, RMSEA = 0.032, SRMR = 0.018), with all standardized factor loadings for each construct beyond 0.70.

We assessed both the convergent validity of each construct and the discriminant validity between them. Convergent validity was determined by calculating the Average Variance Extracted (AVE). To evaluate discriminant validity, we compared the AVE values of each construct against the squared correlations with other constructs. The AVE values were as follows: 0.73 for the attribution of COVID-19 crisis responsibility, 0.73 for trust in government health agencies, 0.82 for anger, and 0.72 for vaccination intention. All values exceeded the acceptable threshold of 0.50, indicating strong convergent validity. Additionally, each construct’s AVE was greater than its squared correlations with the other constructs (see Table 2 for the results of the squared correlations among the latent constructs).

After establishing the measurement model, we tested the full structural equation model with gender, age, education, income, and political stance as exogenous variables. The proposed structural model showed a good fit to data; χ^2^[259] = 881.078, CFI = 0.993, TLI = 0.99, RMSEA = 0.027, and SRMR = 0.017.

### 4.2. Main Analysis

**Figure 2:**
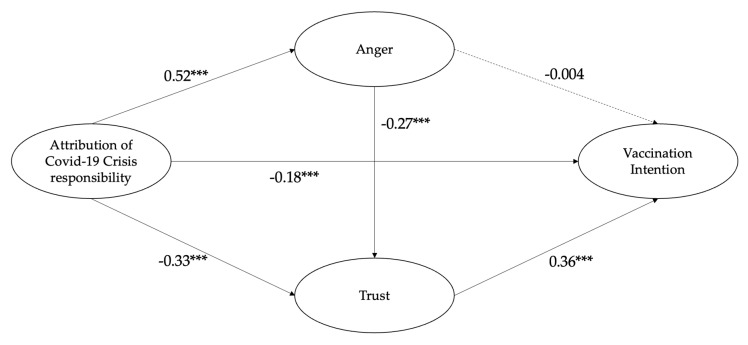
SEM results of the hypothesized model. Note. Model fit: χ^2^/df = 3.40, CFI = 0.993, TLI = 0.990, RMSEA = 0.027, and SRMR = 0.017. Standardized regression coefficients are reported. Control variables include gender, age, education level, monthly household income, and political stance. Covariances between exogenous variables and indicators for latent variables are not shown here in the figure. *** *p* < 0.001.

The standardized path coefficients and the statistical significance for each path are presented in Figure 2. First, we explored whether the attribution of COVID-19 crisis responsibility predicts intention to receive COVID-19 vaccines. We found that the attribution of crisis responsibility was directly and negatively associated with vaccination intention (*β* = −0.18, *p* < 0.001). Hence, a direct path between crisis attribution and vaccination intention was identified.

The results showed that the attribution of COVID-19 crisis responsibility was negatively associated with trust in government health agencies (*β* = −0.33, *p* < 0.001); **H1** was supported. Trust in government health agencies was positively associated with the intention to receive COVID-19 vaccines (*β* = 0.36, *p* < 0.001); **H2** was supported. 

The attribution of COVID-19 crisis responsibility was also found to have a positive relationship with anger (*β* = 0.52, *p* < 0.001), providing support for **H3**. Anger was negatively associated with trust in government health agencies (*β* = −0.27, *p* < 0.001); **H4** was supported. However, anger was not significantly associated with the intention to receive vaccines (*β* = −0.004, *p* > 0.05); Thus, **H5** was not supported.

The indirect paths were examined by a series of bootstrapping analyses with 5000 bootstrapped samples. As shown in Table 3, the results indicate that trust in government health agencies mediated the relationship between the attribution of COVID-19 crisis responsibility and vaccination intention; the attribution of COVID-19 crisis responsibility predicted lower levels of COVID-19 vaccination intention through decreased trust in government health agencies (estimate = −0.14, *SE* = 0.01, 95% CI = [−0.171, −0.117]). Confirming the mediating role of trust, **H6** was supported.

On the other hand, due to the insignificant relationship between anger and vaccination intention, anger did not mediate the relationship between crisis attribution and vaccination intention; **H7** was not supported. 

The serial mediation path via anger and trust was found to be significant; estimate = −0.06, *SE* = 0.01, and 95% CI = [−0.073, −0.048]. The attribution of COVID-19 crisis responsibility predicted lower levels of intention to receive COVID-19 vaccines through greater anger and decreased trust in government health agencies. **H8** was supported. See Table 3 for details of the bootstrap results.

## 5. Discussion

This study examines the pathways through which the attribution of crisis responsibility predicts the public’s decision to accept recommended protective behaviors (i.e., intention to receive COVID-19 vaccines), particularly, by using the case of Hong Kong. First, our findings show that perceived COVID-19 crisis responsibility both directly and indirectly predicts lower vaccination intention. The direct impact of crisis attribution on vaccination intention suggests that the public viewed the COVID-19 pandemic as a preventable crisis and ascribed a higher level of responsibility to the government, hence it hindered their acceptance of protective actions. We also found the mechanism by which crisis attribution hinders the public’s uptake of protective actions. Specifically, the perceived crisis responsibility predicted less willingness to take COVID-19 vaccines via decreased trust. This result demonstrates that greater organizational blame can hamper the acceptance of protective actions by eroding public trust in a responsible organization during health crises.

Furthermore, the results of this study uncovered the affective mechanism through which crisis attribution yields less willingness to accept vaccinations. In line with the previous studies on the impact of crisis emotion on the public’s perception of an organization, our findings revealed that anger aroused by crisis attribution undermines trust in government health agencies [15,16,41]. This, in turn, would prompt less willingness to accept vaccination. In other words, when the government is deemed accountable for the COVID-19 crisis, it evokes greater feelings of anger and thus decreases trust in government health agencies, ultimately serving as an impediment to vaccination. On the other hand, unlike our prediction, anger was not found to affect vaccination intention directly. It indicates that experiencing anger alone fails to motivate people to repudiate protective actions. An insignificant path between anger and vaccination might also have been affected by the direct and strong association between the attribution of COVID-19 crisis responsibility and vaccination intention. While anger and vaccination were negatively correlated in the correlational analysis, their relationship was reduced to insignificance when they were included in the final SEM model. At the same time, the direct and negative association between crisis attribution and vaccination remained significant. Moreover, the additional non-linear regression analysis revealed that the quadratic relationship between anger and vaccination was not significant (*b* = −0.01, *p* = 0.53), which corroborates their insignificant relationship. Our result indicates that the high levels of crisis responsibility placed on the government yielded direct and adverse consequences for the public’s uptake of protective actions. This finding adds a new perspective to understanding the direct and indirect influence of crisis responsibility on pro-health outcomes.

Taken together, our findings highlight not only a direct process in which the perceived crisis responsibility predicts vaccination intention but also the mediation processes in which anger and trust serve as key mediators in this effect. The results of this study provide critical insights into the psychological mechanisms by which the public’s evaluation of crisis responsibility would impact their risk-related judgment and decision to take vaccinations. By illuminating the role of attributed responsibility in affecting vaccine uptake, this study advances the knowledge about how the attribution process during health crises could impact individuals’ willingness to engage in preventive behaviors. Particularly, in terms of investigating the mediational routes, we identified that public trust in an organization was a cornerstone to facilitating vaccination; trust played a crucial role in linking crisis responsibility with vaccination intention and mediated the sequential effect through anger. This study went a step further than prior research that underscores the role of trust in organizational conflicts by identifying and expanding its role as a motivator that drives health decision-making [29,30,31]. As one of the few studies that account for the perception of crisis responsibility as a key antecedent of vaccination, it contributes to enhancing the understanding of what shapes vaccination decisions during public health emergencies by elucidating its mechanisms.

The results of this study are of great value for communication professionals and government authorities working in public health campaigns to foster vaccine advocacy behaviors. Overall, our findings underscore the adverse impact of perceived crisis responsibility on vaccination intention. Hence, it is important to develop communication strategies that reduce the organizational responsibility frame to elicit the public’s support for vaccines. In the case of the COVID-19 pandemic, there was a sheer volume of information that was framed against government agencies, which allocates responsibility and blame for the pandemic crisis [45,46]. In this regard, during global health emergencies, more active communication efforts from an organization’s side are needed to mitigate the impact of such frames; for instance, government agencies should prepare a clear message strategy to address concerns about responsibility frames, and they also need to devise and implement plans to debunk misinformation about crises and vaccines.

In assessing this study’s findings, several limitations should be kept in mind. First, while this study focuses on trust and anger as mediators between responsibility attribution and vaccination intention, other emotions (e.g., fear and anxiety) and cognitive factors (e.g., self-efficacy, vaccine efficacy) could also play significant roles in influencing vaccination intention. Future research could expand the current model by exploring the mediating effects of these additional variables to provide a more comprehensive understanding of the psychological mechanisms involved in public health behaviors during crises. Second, this study used cross-sectional data, which involves difficulties in ensuring the causality in relationships. Future research could benefit from longitudinal data collection, which would allow for tracking changes over time and provide clearer evidence of the temporal order between crisis responsibility attribution and vaccination intention. Additionally, experimental designs that manipulate perceptions of crisis responsibility would offer a more controlled environment to test causal effects and help isolate the impact of this variable on public health behaviors. Third, this study used a single-item scale to measure the attribution of crisis responsibility for the central and Hong Kong SAR governments. While this approach allowed us to capture a general perception of governmental responsibility, it may not fully reflect the complexity of public perceptions regarding different aspects of crisis management. In future research, a more comprehensive multi-item scale could be developed to capture a wider range of dimensions, such as vaccine distribution, anti-pandemic policy, and healthcare management. This would offer a more detailed and nuanced understanding of how responsibility is attributed across various aspects of government action during a public health crisis. Additionally, expanding this scale could improve the reliability and granularity of the measurement, further enhancing the explanatory power of future models. Fourth, the findings of this study were generated from the population of Hong Kong. It is important to test whether the proposed model is generalizable to other countries or regions. Conducting comparative studies across different societal contexts will provide a more nuanced understanding of whether and how the attribution of crisis responsibility could determine vaccination decisions. In addition, future studies could explore how the relationships between crisis responsibility attribution and vaccination intention vary across different demographic subgroups, such as gender, age, political stance, or education level. A multi-group SEM analysis could provide deeper insights into whether these factors moderate the effects observed in the current study. This would help refine our understanding of how diverse populations respond to public health crises and health-protective behaviors. Finally, this study looked into the role of anger as a key affective factor that helps explain the impact of crisis attribution. It is worth noting that there are a range of emotions salient in crises that may trigger different action tendencies [34,42]. Hence, studies could further explore how other discrete emotions (e.g., fear, anxiety, disgust, sadness) play a role in the mechanisms that shape vaccination decisions.

Despite these limitations, this study has theoretical importance due to its identification of the cognitive and affective pathways through which crisis attribution predicts health decision-making [47]. The findings of this study inform further empirical research that investigates the role of the crisis responsibility judgment in shaping vaccine acceptance during public health crises. In addition, our findings offer guidance to public health practitioners and crisis managers on developing effective messaging strategies to deal with the organizational responsibility frames and promote vaccine uptake.

## 6. Conclusions

In conclusion, this study demonstrates that attributing COVID-19 crisis responsibility to the government negatively impacts vaccination intention, both directly and indirectly through reduced trust in government health agencies. While anger arising from crisis attribution decreases trust, it does not directly influence vaccination intention. These findings highlight the importance of managing public perceptions of responsibility and trust in health agencies to promote vaccine uptake. Future research should explore these dynamics in different contexts and investigate the role of other emotions in shaping public health behaviors during crises.

## Figures and Tables

**Table 1:** Sample profiles (*N* = 3188).

Factors	%
**1. Gender ** Female Male	56.6% 43.4%
**2. Age ** 20–24 25–29 30–34 35–39 40–44 45–49 50–54 55 or above	9.1% 11.5% 13.0% 12.9% 11.8% 8.9% 12.0% 20.7%
**3. Education ** Junior high school or below High school Foundation diploma Advanced diploma Bachelor’s degree Master’s degree Doctoral degree	3.5% 25.3% 5.1% 13.2% 40.8% 11.2% 0.9%
**4. Monthly household income ** Under HK$10,000 HK$10,001–HK$20,000 HK$20,001–HK$30,000 HK$30,001–HK$40,000 HK$40,001–HK$50,000 HK$50,001–HK$60,000 Above HK$60,000	3.0% 10.8% 15.1% 19.5% 17.1% 11.9% 22.6%
**5. Political stance ** Pro-Establishment Centrist Democrat Nativist Apolitical Others	10.3% 12.8% 22.9% 10.2% 39.1% 4.8%

**Table 2:** Descriptive statistics and correlation results of the main variables.

Variables	Mean (Standard Deviation)	1	2	3	4
1. Attribution of Covid-19 crisis responsibility	5.29 (1.39)	—			
					
2. Trust in government health agencies	4.05 (1.32)	−0.51 ***	—		
		(0.26)			
3. Anger	4.46 (1.51)	0.56 ***	−0.49 ***	—	
		(0.31)	(0.24)		
4. Vaccination intention	3.88 (2.13)	−0.37 *** (0.14)	0.46 *** (0.22)	−0.29 *** (0.08)	—

Note: The numbers in parentheses indicate the squared correlations between latent constructs. *** *p* < 0.001. The mean and standard deviation scores are composite measures derived from averaging responses across the items assessing each variable (see the “Measures” section for detailed items).

**Table 3:** Bootstrap analyses of the mediating pathways.

			Bias-Corrected 95% CI		
Mediation Path	Bootstrap Estimate	S.E.	Lower	Upper	*p*-Level
Attribution of COVID-19 crisis responsibility → Trust in government health agencies → Vaccination intention	−0.14	0.01	−0.171	−0.117	***
Attribution of COVID-19 crisis responsibility → Anger → Trust in government health agencies → Vaccination intention	−0.06	0.01	−0.073	−0.048	***

Note. *** *p* < 0.001.

## Data Availability

The data are available upon request.
